# Epulis and Myeloid Tumors of the Jaw

**Published:** 1872-07

**Authors:** 


*Epulis and Myeloid Tumors of the Jaw.*—Prof. Gross, speaking of Epulis in a recent clinical lecture remarked that—

"He had never before met with a growth of this kind at so early an age as seven years. It is usually a tumor of slow growth and differing from myeloid in this respect; it is often painful; pntients afflicted with it suffering much from toothache. Again, epulis is often partially osseous, frequently containing spicula of bone in its centre, detached from the surface of the bone. It recurs under the same circumstances with myeloid, that is, when all parts have not been completely extirpated, though perhaps less frequently than in the former. It is generally lobulated, as myeloid tumor in the same situation, but its structure is firmer. It is tougher and more elastic, owing to its fibrous structure. On section of myeloid tumors, more decided characteristics are noticed, which may be recognizable by the naked eye. The cut surfaces are 'smooth, uniform, compact, shining, succulent, with a yellowish, not a creamy fluid;' presenting 'blotches, of dark or livid crimson, or of a brownish or a brigher blood color, or of a pale pink, or all of these tints mingled on the grayish-white or greenish basis-color.' Epulis on section is uniform, firm, white and shining, presenting often in its interior the spicula to which allusion has been already made. Before operation it is not easy to decide whether a tumor is epulis or myeloid, and though appearances on section are more characteristic, they do not become available for diagnosis. As a matter of prognosis it is not of paramount importance that the exact nature of the tumor be known before operation, supposing it one of these two forms, as neither is apt to return if *thoroughly* removed. Recurrence of each occasionally takes place, and it is somewhat more frequent in the case of myeloid; the periosteum should in all instances be scraped after operation. As the only certain means of relief, Prof. Gross recommends 'excision of the piece of bone to which it is attached.'"

